# Health Behavior, Level of Hemoglobin A1c, and Quality of Life Among Agricultural Workers of Various Ethnicities in Thai Border Communities

**DOI:** 10.3389/fmed.2022.796955

**Published:** 2022-02-15

**Authors:** Sorawit Boonyathee, Parichat Ong-Artborirak, Katekaew Seangpraw, Prakasit Tonchoy, Supakan Kantow, Sasivimol Bootsikeaw, Nisarat Auttama, Monchanok Choowanthanapakorn, Dech Dokpuang, Pitakpong Panta

**Affiliations:** ^1^School of Medicine, University of Phayao, Phayao, Thailand; ^2^Faculty of Public Health, Chiang Mai University, Chiang Mai, Thailand; ^3^School of Public Health, University of Phayao, Phayao, Thailand; ^4^School of Allied Health Sciences, University of Phayao, Phayao, Thailand; ^5^School of Nursing, University of Phayao, Phayao, Thailand

**Keywords:** healthcare behavioral, quality of life, hemoglobin A1c, agricultural workers, ethnicities

## Abstract

**Background:**

Little is known about the glycated hemoglobin (HbA1c) levels and quality of life (QoL) in ethnic minority agricultural workers. We investigated the links among health behaviors, HbA1c levels, and QoL ethnic agricultural workers living in rural areas.

**Methods:**

A cross-sectional study was conducted in three northern Provinces of Thailand. Agricultural workers of Indigenous, Hmong, Karen, and Lua communities were recruited. The number of 468 samples were selected using multistage sampling. Data collection was done from interviews using questionnaires, and blood samples were taken.

**Results:**

We revealed 56.8% of participants to be female, with an average age of 49.6 years. Also, 56.0 and 34.8% of participants had low and moderate levels of knowledge on non-communicable diseases, respectively. In addition, 56.8 and 30.6% of participants had moderate and low health behaviors, respectively. Also, 51.5% had a HbA1c level (≥6.5%). We found that 64.7, 22.9, and 12.4% had moderate, low, and high QoL, respectively. Multiple linear regression analysis revealed that having an underlying disease and knowledge score were both significantly related to the health behaviors score (*p* < 0.05), accounting for 68.6% of the variance. Five variables (ethnicity, BMI, having an underlying disease, smoking, and health behaviors) were significantly related to the HbA1c level (*p* < 0.05), accounting for 24.6% of the variance. Education, health behaviors, and HbA1c level were significantly associated with QoL (*p* < 0.05). These three factors could explain 79.4% of the variance in QoL among ethnic agricultural workers.

**Conclusion:**

Health behaviors of ethnic minority agricultural workers influenced their HbA1c level and QoL. Effective health behaviors modification programs should be developed in accordance with the problems and needs among ethnic minority agricultural workers to enhance their QoL.

## Introduction

Agriculture has the largest proportion of informal employment, which is estimated to be more than 90% (1). Most developing countries, like Thailand, have a significant informal sector. According to the formal employment survey 2020, more than half of Thai workers were informal workers (53.8%), with 55.6% working in the agricultural sector (2). Agricultural workers in the informal sector often have low wages and working conditions (3–5). In addition, the majority of Thailand's informal workers are ethnic minority groups residing in rural areas. These ethnic minorities have migrated from neighboring countries. They are distributed in the north provinces, such as Chiang Rai, Phayao, Lamphun, Nan, and Tak (6, 7). Thailand has an estimated population of over 4 million migrants, but no valid population-based data on their health status exists (8).

Most of these individuals are living in poor economic conditions (7–10). For most of their lives, they are engaged in agricultural activities. Some are employed as “general labor” on the highlands, such as growing rice, and planting local vegetables along the hillside. According to one survey, most of these ethnic minorities are informal workers without a Thai national identification card, so they cannot apply for better-paid jobs in the lowlands or cities (11, 12). Some of these ethnic minorities must live in a forest to find rare products to sell for a living. Previously, their lives were unstable because they had to migrate to a better place to live and to obtain better-paid employment (11, 12). They continue to experience problems in daily life, such as having poor access to health services, or being unable to access to certain public services and/or education (11). The neglect of ethnic minorities could create health consequences for them especially epidemics and chronic non-communicable diseases (11–13) which, ultimately, will affect the economy, society, and public-health system of Thailand (13).

Ethnic minorities are designated “vulnerable populations” with a range of health concerns, including non-communicable diseases (NCDs) (e.g., hypertension, diabetes mellitus (DM), hyperlipidemia) due to their specific lifestyles, cultures and diets (9–11). One prospective study indicated that a group of farmers exposed to certain pesticides carried a higher risk of developing DM (14). One study indicated that DM incidence among Thai farmers was associated with pesticide exposure from their working environment (15). Several studies have found that hazardous substances from pesticides can affect insulin secretion, glucose homeostasis, and other related symptoms such physiological, stress, and oxidative (16–19). These factors, which include health behaviors and agricultural activities, may have an impact on the health and quality of life (QoL) of ethnic minority agricultural workers, who contribute economic value for a country. In this study, we focused on their HbA1C levels, which can be useful for people with undiagnosed type 2 diabetes.

QoL studies have been conducted extensively in the field of medicine and public health (20). Those studies have explored the factors influencing the QoL and impact of certain diseases and treatment on the QoL among patients and health-risk groups (9, 10, 20, 21). The Quality of Life Report mentioned that QoL quantifies health status as a component related to physical functioning as well as mental, emotional, and social conditions (21). The concept goes beyond direct measures of population health, life expectancy, and causes of death, and focuses on the impact that health status has on the QoL (21). An individual's life experience is dependent upon the goals, expectations, standards, and concerns in different cultures and value systems (20, 21).

The literature suggests that the QoL among agricultural workers should be improved, and that attention must be given to all dimensions of QoL components, especially for workers in rural areas (21). Health and QoL among informal workers differ on the basis of knowledge and experience that affects their lifestyles (22). Some studies have suggested that personal characteristics, suffering from DM, and self-care behaviors can affect (directly or indirectly) the level of HbA1c and QoL of informal workers (23). One literature review showed that the key factors influencing levels of blood sugar and lipids were age, sex, knowledge and perception of health status, and health behaviors (e.g., diet, exercise, and emotional management); all of these factors could determine good health and QoL among people (15, 24, 25). Research that links DM and ethnic minority agriculture workers is relatively rare (23–25). Few studies have used this method to assess the QoL and health status among ethnic minorities in northern Thailand (23–25). We were interested in examining knowledge, health behaviors, HbA1c levels, and QoL among ethnic minority agriculture workers in the upper and rural areas of northern Thailand, as well as the relationships among these factors. In concept, health behaviors are considered to influence both HbA1c levels and quality of life, and HbA1c levels may also play a role in quality of life. The results of this study could be used to initiate health-promotion programs to control an abnormality of blood glucose levels and to prevent non-communicable diseases and health complications. However, the programs should be appropriated in the context of local rural communities and ethnic minorities.

## Methods

**Figure 1:**
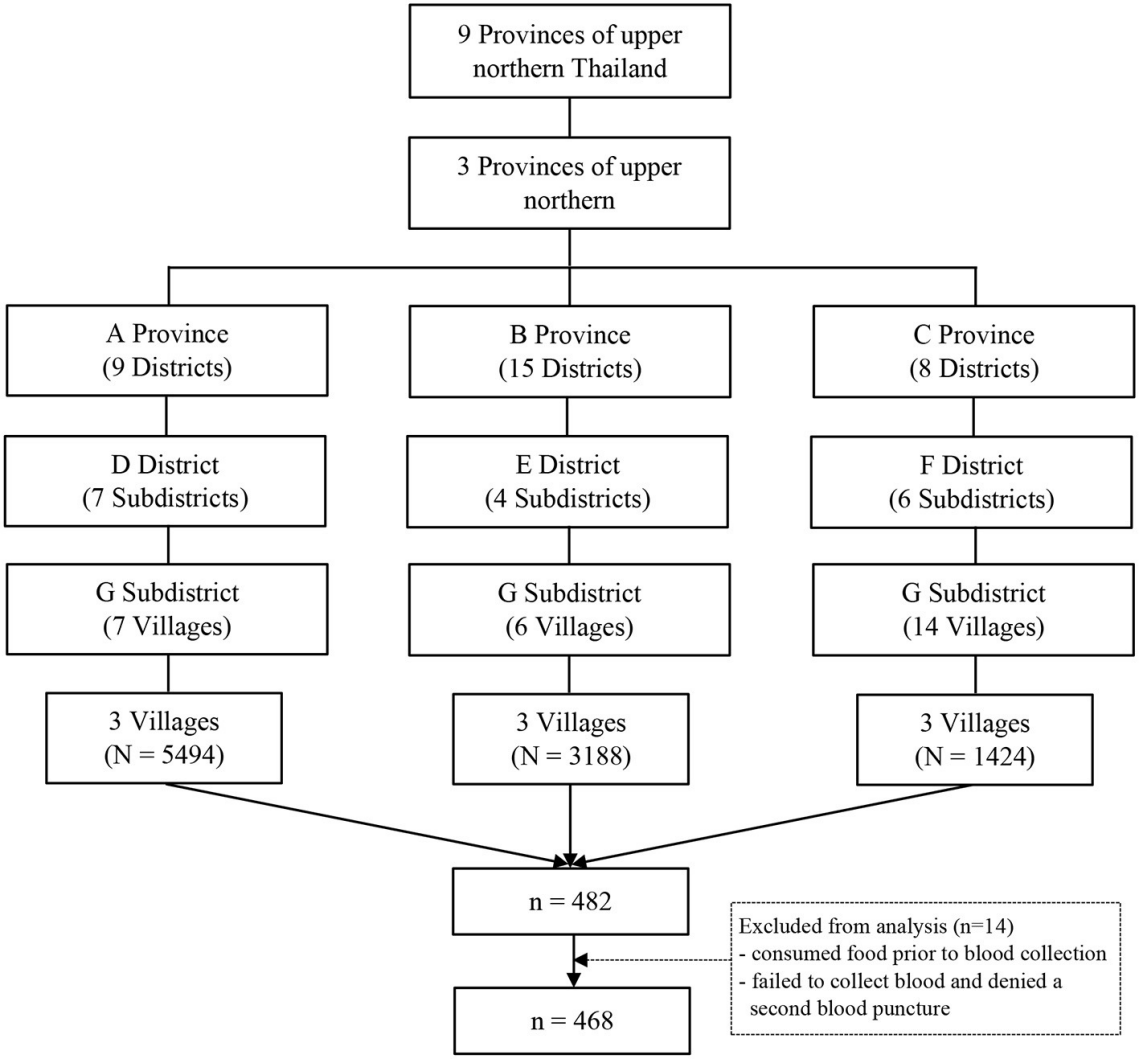
Flow diagram of participants.

This research was a cross-sectional study under the Unit of Excellence “Health Promotion and Quality of Life.” Data were collected from November 2020 to April 2021 in the northern border provinces where highland and lowland ethnic minority groups live, which comprises the provinces of Lamphun, Phayao, and Nan. Nan Province is a highly mountainous area located in the easternmost part of northern Thailand, near the Laos border. Phayao Province is a plateau area surrounded by mountains, and is adjacent to Nan Province and Laos (26). Lamphun Province is the smallest province located in the north. The geographical area is flat and it is far from the capital city (26). Previously, many ethnic minority groups migrated from Myanmar and Laos to settle in large numbers in northern Thailand (27). Large populations of ethnic minorities were living in the three Provinces we selected. The study areas are about 30–40 km from city centers, and access to public services (including primary care centers and Tambon Health Promoting Hospitals) is difficult. The study cohorts from each Province were selected using a multistage sampling method (Figure 1). First, the three Provinces were selected. Second, we selected districts using a simple random sampling method by lottery, resulting in one district per province. Third, three sub-districts were chosen randomly from each district. Each of the three villages in each sub-district was selected randomly. Then, the recruitment of volunteers to participate in our study was announced through public relations using health volunteers, the head of the village, and health workers in the primary care unit as the main communication channels. The samples were selected using simple random sampling. The sample size was calculated using Cochran's formula with a proportion of 50% (assuming maximum variability), precision of 5%, and 95% confidence level (28). We allowed for a dropout prevalence among participants of 25%. We enlisted the participation of 482 people.

The inclusion criteria were females and males: (a) aged ≥20 years; (b) being an ethnic minority registered with the Health Center of Ethnic Group Migrant Peoples, and Migrant Workers, Department of Health, Ministry of Public Health and municipalities in the area; (c) residing in the area for ≥2 years to avoid enlisting temporary migrant workers; (d) able to communicate in the local language; (e) who provided written informed consent before study initiation. People with cognitive and/or psychological disorders, or with gestational diabetes, were excluded from the study, as determined by a research assistant who was familiar with the local population.

Most potential recruits did not speak Thai. Therefore, to obtain correct data, we recruited research assistants from the study area to communicate appropriately and correctly. Before study initiation, an announcement was made, and we recruited 15 research assistants from each sub-district: three public-health scholars, two nurses, and 10 health volunteers from the village. They were able to communicate in the local language and to access study participants (hereafter termed “participants”) rapidly and conveniently.

Before data collection, a meeting for research assistants in each area was held to clarify the objectives of the research, data-collection process, understanding of questionnaires, and the privacy of participants. We aimed to ensure that all research assistants understood the process, interview method, and research details. The study was conducted during the “second wave” of coronavirus disease 2019 (COVID-19) epidemic, so the procedure was authorized by the local government agency and head of the village. All procedures for data collection followed the government measures for COVID-19 prevention which was announced in each community area. The head of the village announced a review of the research to clarify the objectives and necessary background information through the village radio broadcast in the morning using the local dialect.

After receiving the signed consent forms, a blood specimen (3 ml) was taken for HbA1c analysis. The latter were interviewed face-to-face from 9 am to noon in a private room in the village with the help of a research assistant. The latter interviewed and allowed the participants to respond to questions. Each interview lasted approximately 15–20 min per person. Trained physicians and family care teams undertook the initial physical examinations of all participants in an appropriate room provided by the community. A small gift was given to each participant after completing the survey.

The research instrument used to collect quantitative data was developed to be suitable for people living in rural areas, and comprised four parts. Part 1 focused on general demographic characteristics: sex, age, ethnicity, marital status, education, and income. The health assessment comprised underlying disease, body mass index (BMI), alcohol intake, and tobacco smoking. Part 2 centered on a knowledge questionnaire on NCDs. It comprised 10 items adapted to be suitable for people living in rural areas (29). The questions were multiple-choice and allowed the participant to respond with a correct or incorrect answer. The total score was in the range 0–10 points. The total score was divided into three levels: “high” (≥8 points), “moderate” (6–7 points), and “low” (≤5 points). Part 3 focused on a questionnaire on health behaviors applicable for research in rural areas (29). It consisted of questions on general health behaviors, such as food consumption and exercise. The questionnaire comprised 12 items. The questionnaire was a rating scale with three levels: “never practiced,” “rarely practiced” (1–3 times/week), and “regularly practiced” (4–6 times/week). They were divided into three levels: “high” (score ≥80%, i.e., ≥28 points); “moderate” (score of 60–79%, i.e., 21–27 points), and “low” (score <60%, i.e., ≤ 20 points). Part 4 centered on the World Health Organization Quality of Life: Brief Version (WHOQOL–BREF) (30). We employed the version developed by the WHO translated for Thailand in a short form (WHOQOL–BREF–THAI) (31, 32). The latter consisted of 26 items divided into four domains: Physical Health with seven items (DOM1); Psychological Health with six items (DOM2); Social Relationships with three items (DOM3); Environmental Health with eight items (DOM4). Each item of WHOQOL–BREF (30) is scored from 1 to 5 on a response scale. The scores were divided into three levels: a score of 26–60 denoted a “poor” QoL; a score of 61–95 reflected a “moderate” QoL; a score of 96–130 indicated a “good” QoL.

An analyzer was used to measure the HbA1c level among participants. A medical technician from the School of Allied Health Sciences within the University of Phayao (Thailand) undertook the collection and analyses of blood, and interpretation of results. The laboratory equipment passed the quality inspection required.

All questionnaires were developed based on the literature. After completing the first draft of the questionnaires, they were checked for accuracy using the Item Objective Congruence (IOC) method. Then, they were checked by external experts in their respective fields (internal medicine, behavioral health, public health). A question with a score <0.5 was eliminated from the set of questions. A question with a score 0.5–0.69 was revised based on feedback from experts. A question with a score >0.7 was considered to be acceptable for use in data collection. The questionnaire was tested on 30 participants with similar characteristics. For the knowledge questionnaire (Part 2 of the research instrument), the Kuder Richardson formula (KR20) = 0.80. Part 3 and Part 4 of the research instrument were carried out to check the reliability of the questionnaire using Cronbach's alpha coefficient: the value was 0.79 and 0.84, respectively.

### Statistical Analysis

Statistical analyses were undertaken using SPSS 17 (IBM, Armonk, IL, USA) licensed from Chiang Mai University (Thailand). General information was described using mean, standard deviation (SD), minimum (Min), and maximum (Max) values, as well as frequencies and percentages. We used linear regression analysis to examine the factors related to health behaviors, HbA1c level, and QoL in ethnic minority agricultural workers. After the univariable analysis, variables with a *p* < 0.15 were analyzed together in a multivariable model using the enter technique. The final model in which all predictors were found to be significant at *p* < 0.05 is presented. The unstandardized coefficient (B) and standardized coefficient (Beta) are reported.

## Results

### Sociodemographic Characteristics

Table 1 presents the sociodemographic characteristics of participants categorized based on minority group. The mean age of the participants was 49.6 years (SD = 13.8). Ethnic minorities were classified as Hmong (50.6%), Lua (23.0%), Indigenous (15.2%), and Karen (11.2%). We found that 56.8% of participants were female, 52.1% did not have an educational background, and 49.6% were financially insufficient. We discovered that 50.6% consumed alcohol and 73.1% did not smoke cigarettes. We revealed that 58.5% had an underlying disease diagnosed by a doctor, such as hypertension (39.7%), hyperlipidemia (7.6%), DM (5.8%), stroke (3.0%), heart disease (1.7%), or kidney disease (0.4%). The average BMI of the study was 24.7 kg/m^2^ (SD = 4.5).

**Table 1:** Sociodemographic of participants categorized based on minority groups.

**Variables**	**Overall** **(*n* = 468)**	**Indigenous** **(*n* = 71)**	**Hmong** **(*n* = 237)**	**Karen** **(*n* = 52)**	**Lua** **(*n* = 108)**
	***n* (%)**	***n* (%)**	***n* (%)**	***n* (%)**	***n* (%)**
Gender					
Male	200 (43.2)	29 (40.8)	112 (47.3)	26 (50.0)	35 (32.4)
Female	266 (56.8)	42 (59.2)	125 (52.7)	26 (50.0)	73 (67.6)
Age (years)					
Mean ± SD	49.6 ± 13.8	59.8 ± 10.5	46.8 ± 12.9	59.1 ± 13.2	44.2 ± 12.0
Min–Max	20–89	36–88	20–89	20–79	22–89
Marital status					
Single/widowed/separated	142 (30.3)	36 (50.7)	49 (20.7)	13 (25.0)	44 (70.0)
Married	326 (69.7)	35 (49.3)	188 (79.3)	39 (75.0)	64 (59.3)
Education					
No	244 (52.1)	50 (70.4)	129 (50.4)	24 (46.2)	41 (38.0)
Yes	224 (47.9)	21 (29.6)	108 (45.6)	28 (53.8)	67 (62.0)
Financial status					
Insufficient	232 (49.6)	42 (59.2)	120 (50.6)	34 (65.4)	36 (33.3)
Sufficient	236 (50.4)	29 (40.8)	117 (49.4)	18 (34.6)	72 (66.7)
Alcohol consumption					
No	231 (49.4)	51 (71.8)	117(49.4)	26 (50.0)	37 (34.3)
Yes	237 (50.6)	20 (28.2)	120(50.6)	26 (50.0)	71 (65.7)
Smoking status					
Non-smoker	342 (73.1)	51 (71.8)	164 (69.2)	39 (75.0)	88 (81.5)
Smoker	126 (26.9)	20 (28.2)	73 (30.8)	13 (25.0)	20 (18.5)
Body mass index (kg/m^2^)					
Mean ± SD	24.7 ± 4.5	22.8 ± 3.7	24.3 ± 3.7	30.7 ± 4.2	23.8 ± 4.3
Min–Max	15.2–42.0	16.6–36.8	16.0–36.2	22.2–42.0	15.2–37.8
Current disease					
No	194 (41.5)	19 (26.8)	117 (49.4)	21 (40.4)	37 (34.3)
Yes	274 (58.5)	52 (73.2)	120 (50.6)	31 (59.6)	71 (65.7)
Type of disease					
Hypertension	186 (39.7)	39 (54.9)	77 (32.5)	21 (40.4)	49 (45.4)
DM	27 (5.8)	2 (2.8)	12 (5.1)	6 (11.5)	7 (6.4)
Stroke	14 (3.0)	5 (7.0)	4 (1.7)	3 (5.8)	2 (1.9)
Hyperlipidemias	36 (7.6)	6 (8.5)	19 (8.0)	1 (1.9)	10 (9.2)
Kidney	4 (0.9)	0 (0.0)	2 (0.8)	0 (0.0)	2 (1.9)
Heart	7 (1.5)	0 (0.0)	6 (2.5)	0 (0.0)	1 (0.9)

### Knowledge, Health Behaviors, HbA1c Level, and Quality of Life Among Participants

The score for knowledge ranged from 3 to 9, with a mean of 5.4 (SD = 1.2) (Table 2). We discovered that 56.0% of participants had a low level of knowledge, followed by 34.8% who had a moderate level, and 9.2% had a high level of knowledge. The highest mean scores for knowledge were found in Lua, followed by Hmong, Karen, and Indigenous people. Another interesting variable about the scores of health behavioral ranged from 18 to 30 (mean = 22.7, SD = 2.9). With regard to health behavioral, 56.8% had a moderate level, 30.6% had a low level, and 12.6% had a high level. The highest mean scores for health behaviors were found in Lua, followed by Hmong, Karen, and Indigenous people. Results revealed that 51.5% of participants had HbA1c ≥6.5, which was interpreted as an abnormality. Indigenous people had the highest mean HbA1c levels, followed by Lua, Hmong, and Karen. The QoL score among participants was 56–103 (mean = 72.3, SD = 12.5). We discovered that 64.7% of participants had a moderate level of QoL, 22.9% had a low level, and 12.4% had a high level. The highest mean scores for QoL were found in Lua, followed by Hmong, Karen, and Indigenous people.

**Table 2:** Descriptive analysis of knowledge, healthcare behaviors, HbA1c, and quality of life among participants.

**Variables**	**Overall** **(*n* = 468)**	**Indigenous** **(*n* = 71)**	**Hmong** **(*n* = 237)**	**Karen** **(*n* = 52)**	**Lua** **(*n* = 108)**
	***n* (%)**	***n* (%)**	***n* (%)**	***n* (%)**	***n* (%)**
Knowledge					
Low level (scores ≤ 5)	262 (56.0)	54 (76.1)	140 (59.1)	31 (59.6)	37 (34.3)
Moderate level (scores 6–7)	163 (34.8)	16 (22.5)	73 (30.8)	15 (28.8)	59 (54.6)
High level (scores ≥8)	43 (9.2)	1 (1.4)	24 (10.1)	6 (11.5)	12 (11.1)
Mean ± SD	5.4 ± 1.2	4.9 ± 0.9	5.39 ± 1.2	5.2 ± 1.4	5.9 ± 1.2
Min–Max	3–9	3–8	3–9	3–8	3–9
Health behavior					
Low level (scores ≤ 20)	143 (30.6)	30 (42.3)	77 (32.5)	26 (50.0)	10 (9.3)
Moderate level (scores 21–27)	266 (56.8)	38 (53.5)	128 (54.0)	18 (34.6)	82 (75.9)
High level (scores ≥28)	59 (12.6)	3 (4.2)	32 (13.5)	8 (15.4)	16 (14.8)
Mean ± SD	22.7 ± 2.9	21.5 ± 2.0	22.7 ± 3.0	22.3 ± 3.3	23.8 ± 2.5
Min–Max	18–30	19–29	19–31	18–30	19–30
HbA1c (%)					
Normal (<6.5)	227 (48.5)	37 (52.1)	109 (46.0)	31 (59.6)	50 (46.3)
Abnormal (≥6.5)	241 (51.5)	34 (47.9)	128 (54.0)	21 (40.4)	58 (53.7)
Mean ± SD	6.6 ± 1.3	6.8 ± 1.7	6.6 ± 1.1	6.4 ± 1.1	6.6 ± 1.2
Min–Max	3.9–13.8	4.0–13.8	3.9–11.9	4.8–10.6	3.9–11.4
Quality of life					
Low level (scores 26–60)	107 (22.9)	21 (29.6)	61 (25.7)	17 (32.7)	8 (7.4)
Moderate level (scores 61–95)	303 (64.7)	49 (69.0)	142 (59.9)	28 (53.8)	84 (77.8)
High level (scores 96–130)	58 (12.4)	1 (1.4)	34 (14.3)	7 (13.5)	16 (14.8)
Mean ± SD	72.3 ± 12.5	67.2 ± 8.0	72.0 ± 12.9	71.8 ± 13.4	76.8 ± 12.2
Min - Max	56–103	58–100	56–102	56–102	59–103
Physical health					
Mean ± SD	18.7 ± 2.7	18.1 ± 2.1	18.6 ± 2.7	18.5 ± 2.7	19.7 ± 2.8
Min–Max	13.0–30.0	14.0–25.0	13.0–30.0	14.0–26.0	15.0–27.0
Psychological health					
Mean ± SD	16.1 ± 3.2	15.9 ± 2.2	15.7 ± 3.6	15.7 ± 3.2	17.2 ± 2.4
Min–Max	8.0–28.0	11.0–24.0	8.0–27.0	12.0–27.0	13.0–28.0
Social relationships					
Mean ± SD	8.4 ± 2.2	7.6 ± 1.7	8.5 ± 2.4	7.9 ± 2.2	8.7 ± 2.0
Min–Max	3.0–16.0	3.0–12.0	3.0–16.0	5.0–13.0	6.0–15.0
Environmental health					
Mean ± SD	32.0 ± 5.8	20.1 ± 4.2	23.1 ± 5.3	22.6 ± 7.0	24.9 ± 6.4
Min–Max	13.0–40.0	16.0–30.0	15.0–40.0	13.0–40.0	16.0–40.0

### Factors Associated With the Health Behaviors, HbA1c Level, and Quality of Life of Ethnic Minority Agricultural Workers

A strong positive correlation was observed between the score for knowledge on NCDs and health behaviors among ethnic minority agricultural workers (beta = 0.818), which accounted for 66.8% of the variance (Table 3). Multivariable analysis revealed that having an underlying disease and a score of knowledge about NCDs were both significantly related to the score for health behaviors (*p* < 0.05, R^2^ = 68.6%). The HbA1c level was inversely correlated with the score for knowledge (beta = −0.191) and score for health behaviors (beta = −0.305) (Table 4). The final model showed five variables (ethnicity, BMI, having an underlying disease, smoking, health behaviors) to be significantly related to the HbA1c level (*p* < 0.05, R^2^ = 24.6%). Health behaviors could explain 9.1% of the variance in the HbA1c level. The factors influencing the QoL in ethnic minority agricultural workers are shown in Table 5. In the multivariable model, education, health behaviors, and HbA1c level were significantly related to QoL (*p* < 0.05). These three factors accounted for 79.4% of the variance in the QoL. The single factors of health behaviors and HbA1c level accounted for 78.5 and 11.1% of the variance in the QoL, respectively.

**Table 3:** Factors associated with health behaviors in agricultural workers by linear regression (*n* = 468).

**Factors**	**Univariable**			**Multivariable**		
	**B**	**Beta**	***P*-value**	**B**	**Beta**	***P*-value**
Constant				13.38		<0.001^*^
Ethnics					
- Indigenous	Ref.					
- Hmong	1.27	0.218	0.001^*^			
- Karen	0.88	0.095	0.089			
- Lua	2.40	0.346	<0.001^*^			
Gender (female)	0.75	0.127	0.006^*^			
Age (years)	−0.10	−0.467	<0.001^*^			
Education (yes)	3.05	0.523	<0.001^*^			
Marital status (married)	0.42	0.067	0.148			
Financial status (sufficient)	1.73	0.297	<0.001^*^			
BMI (kg/m^2^)	0.01	0.011	0.819			
Current disease (yes)	−1.97	−0.332	<0.001^*^	−0.81	−0.136	<0.001^*^
Alcohol drinking (yes)	0.19	0.033	0.473			
Smoking (yes)	−0.77	−0.117	0.011^*^			
Knowledge (scores)	1.89	0.818	<0.001^*^	1.81	0.783	<0.001^*^
				**R**^**2**^ = 68.6%		

*B, Unstandardized coefficient; Beta, Standardized coefficients*.

* *Significance at the 0.05 level (2-tailed)*.

**Table 4:** Factors associated with HbA1c in agricultural workers by linear regression (*n* = 468).

**Factors**	**Univariable**			**Multivariable**		
	**B**	**Beta**	***P*-value**	**B**	**Beta**	***P*-value**
Constant				6.78		<0.001^*^
Ethnics					
- Indigenous	Ref.			Ref.		
- Hmong	−0.17	−0.069	0.311	−0.05	−0.020	0.739
- Karen	−0.41	−0.102	0.075	−0.83	−0.206	<0.001^*^
- Lua	−0.15	−0.051	0.429	0.11	0.037	0.528
Gender (female)	0.02	0.009	0.840			
Age (years)	0.01	0.053	0.251			
Education (yes)	−0.27	−0.105	0.023^*^			
Marital status (married)	−0.27	−0.099	0.033^*^			
Financial status (sufficient)	−0.10	−0.040	0.388			
BMI (kg/m^2^)	0.05	0.186	<0.001^*^	0.08	0.269	<0.001^*^
Current disease (yes)	0.88	0.343	<0.001^*^	0.52	0.204	<0.001^*^
Alcohol drinking (yes)	0.48	0.191	<0.001^*^			
Smoking (yes)	0.64	0.224	<0.001^*^	0.50	0.177	<0.001^*^
Knowledge (scores)	−0.19	−0.191	<0.001^*^			
Health behaviors (scores)	−0.13	−0.305	<0.001^*^	−0.10	−0.238	<0.001^*^
				**R**^**2**^ = 24.6%		

* *Significance at the 0.05 level (2-tailed)*.

**Table 5:** Factors associated with QoL in agricultural workers by linear regression (*n* = 468).

**Factors**	**Univariable**			**Multivariable**		
	**B**	**Beta**	***P*-value**	**B**	**Beta**	***P*-value**
Constant				−3.67		0.245
Ethnics					
- Indigenous	Ref.					
- Hmong	4.83	0.194	0.004^*^			
- Karen	4.64	0.117	0.038^*^			
- Lua	9.64	0.325	<0.001^*^			
Gender (female)	3.52	0.140	0.002^*^			
Age (years)	−0.36	−0.399	<0.001^*^			
Education (yes)	12.88	0.515	<0.001^*^	1.92	0.077	0.002^*^
Marital status (married)	1.58	0.058	0.208			
Financial status (sufficient)	6.40	0.256	<0.001^*^			
BMI (kg/m^2^)	−0.02	−0.008	0.856			
Current disease (yes)	−8.27	−0.326	<0.001^*^			
Alcohol drinking (yes)	0.82	0.033	0.476			
Smoking (yes)	−3.30	−0.117	0.011^*^			
Knowledge (scores)	7.63	0.770	<0.001^*^			
Health behaviors (scores)	3.79	0.886	<0.001^*^	3.52	0.822	<0.001^*^
HbA1c (%)	−3.31	0.336	<0.001^*^	−0.76	−0.077	0.001^*^
				**R**^**2**^ = 79.4%		

* *Significance at the 0.05 level (2-tailed)*.

In addition, the ethnic groups of Lua and Hmong are analyzed separately. In the ethnic group of Lua, the final model revealed that having an underlying disease and a score of knowledge about NCDs were both significantly related to the score for health behaviors (*p* < 0.05, R^2^ = 47.5%). BMI and health behaviors were related to the HbA1c level (*p* < 0.05, R^2^ = 19.8%). Alcohol consumption, health behaviors, and HbA1c level were significantly related to QoL (*p* < 0.05, R^2^ = 68.9%). In the ethnic group of Hmong, not only having an underlying disease and a score of knowledge about NCDs, but also smoking were significantly associated with the score for health behaviors (*p* < 0.05, R^2^ = 79.6%). Four variables - BMI, having an underlying disease, smoking, and health behaviors - were associated with the HbA1c level (*p* < 0.05, R^2^ = 23.2%). Sex, age, financial status, health behaviors, and HbA1c level were all found to have a significant association with QoL (*p* < 0.05, R^2^ = 83.4%). Due to the small sample size, the association was not examined in Karen and Indigenous people.

## Discussion

This is the first study to link the health behaviors, HbA1c level, and QoL of ethnic minority agricultural workers. We discovered that 51.5% of participants had a high level of HbA1c (>6.5). The effects of poor regulation of the HbA1c level on the body have been discussed extensively (23, 33, 34). Most participants in our study had a low educational level and insufficient income, which may have had a negative impact on their health behaviors. Interestingly, the participants with high HbA1C did not report a history of diabetes because they had not previously or for many years undergone medical examinations and/or health assessments. This phenomenon is similar to that described, who mentioned that uninformed people can make inappropriate judgments toward healthcare behaviors (33). Consistent with the results of a study, we found that participants had limited access to health services which prevented them from being aware of their health status (34). However, such participants were living in a traditional way of life (9, 33). They are unaware of making the correct food choices, which resulted in inappropriate healthcare behaviors in terms of diet. Furthermore, most of their cooking recipes were derived from China and cultural traditions, so some dishes were unhealthy because of extensive use of salt, monosodium glutamate, and oils from animals (33, 35). Similar to a previous showed that hill tribes had a higher prevalence of abnormal HbA1c level (33). One study focusing on African Americans and Latinos showed that both groups had a higher average HbA1c level than that of other ethnic groups; Hispanics had the highest HbA1c level (8.5%) (36). Consistent with the previous studies showed that ethnic minority participants had a high mean level of HbA1c (7.39 ± 1.11%) (23) and some studies showed that participants in Taiwan had a higher mean level of HbA1c (7.8 ± 1.5%) (37).

We found that 56.0% had a low and 34.8% had a moderate level of knowledge. These findings can be explained by the low level of education of participants. In addition, communication between personnel in health departments of hospitals and ethnic minority agricultural workers in each area may be the cause. Most participants used the local dialect, so language could have been a barrier in healthcare communication. This explanation is consistent with a study which stated that language differences are major problems for accessing health services (9). A previous study showed that people who obtained incorrect knowledge toward diabetes did so due to language differences (38). It is consistent with the study which showed one group of people did not have sufficient knowledge to take care of their own health due to lack of communication skills, which prevented them from accessing healthcare information and managing their diabetes (35).

We showed that 64.7% of participants had a moderate QoL score and 22.9% had a low QoL score. During our study, the second wave of the COVID-19 epidemic arose, which impacted on the physical and mental health of participants (2), including anxiety due to reduced income from a lack of work. Quality of life is determined by an individual's satisfaction toward domains such as social relationships, environmental health, and mental health (39). Similar to the concept of Pender's, in which perception of individual toward health status are according to their experience and perception of one's health; which all of these are linked to self-inflicted illnesses (40). Almost half of participants had an underlying disease, which could further reduce their QoL. Some studies have shown that having high blood sugar levels have a significant effect on the QoL of study participants (41). Distress and dissatisfaction with life due to diabetes have been significantly associated with QoL (42).

We discovered that 56.8% had moderate and 30.6% had low health behaviors, which may have been due to most of the participants being uneducated and middle-aged. Pender stated that people with a high level of education are more likely to seek health benefits than those with a low level of education (40). Moreover, the main motivating factor for behavior modification is cognitive, such as specific feelings toward self-care behavior (40). The way a person can change toward healthier healthcare behaviors is due to his/her experiences and perceptions (40). In addition, the literature suggests that healthcare behaviors among ethnic groups are derived from indigenous knowledge, social factors, cultural adherence to self-care plans, self-treatment by “folk” and herbal formulations, and worship of sacred deities to help them heal from their illness (9, 43). In addition, occupational risk-based health conditions are important (44). Similar with the previous study, it was found health behaviors among ethnic minorities to be in accordance with the beliefs, traditions, cultures, and lifestyles of each ethnic group (9). Perceived health status has been shown to be positively correlated with health behaviors at 0.01 level (45). Procedural multiple regression analysis showed that perceptions of health status and attitudes were significant for predicting health-enhancing behaviors among older workers (46).

When analyzing the many variables related to health behaviors, we showed that Hmong and Lua communities had better health behaviors than those of Indigenous people. This was because the health behaviors and practices of each ethnicity were culturally different (9). The Hmong and Lua ethnic groups live in the highlands, and their healthcare behaviors are based on their ancestors. Therefore, these ethnic groups gained experiences from culture, traditions, beliefs, local rituals, and illness to practice in self-care (47). The Indigenous group inhabited flat, plain areas. They began to harmonize the values, social and economic changes in the context of that area (47).

Sex, age, education, income sufficiency, underlying diseases, smoking, and knowledge were significantly associated with health behaviors. Multivariable analysis showed that participants with underlying disease and knowledge on NCDs could predict health behaviors. This finding is consistent with work indicating that poorly literacy can lead to a reduction in health behaviors and healthcare practices; and ethnic minorities are more likely to suffer health complications and to have limited access to healthcare services, which impacts on the quality of their healthcare (48). It is consistent with the study which found that sex, education, economic status, and health status were major predictors associated with deterioration of health and QoL among patients (49). One systematic review showed that several factors contributing to health inequalities among ethnic minorities (including low health literacy and low socioeconomic status) could lead to a higher incidence of illness (50). Similar to a previous study, we found that self-care behavior was an important factor associated with QoL among older ethnic minorities (9).

Studies have suggested that a high blood sugar level is an independent predictor of cardiovascular disease regardless of the diagnosis of DM (50–52). One study suggested that the perception of disease and self-care behaviors were predictive factors significantly associated with the HbA1c level (36). A study from India reported people with a low education level to be at greater risk of having hypertriglyceridemia than those with a higher level of education (53). They also pointed out that smoking is a risk factor for cardiovascular disease (53). We showed that the Karen community had lower levels of HbA1c compared with those of the Indigenous group, which is similar to a study showing that racial/ethnic differences can result in higher triglyceride levels (35). Studies have shown that certain tribes from Lua and Hmong communities carried a significantly higher risk of coronary artery disease compared with that of other ethnic groups, which suggests that differences in racial/ethnic backgrounds can affect triglyceride levels (33). One study undertaken in a large sample in the USA in 2014, it showed that the samples aged 35 and older of different ethnicities had a significantly higher rates of hypertriglyceridemia differently (54).

Multivariable analyses showed that ethnicity, BMI, underlying disease, smoking, and health behaviors were significantly related to the HbA1c level. These data indicated that lifestyle factors, such as obesity, congenital disease, and smoking, were predictors of health problems (55). Several studies have shown that a poor lifestyle and inappropriate healthcare behaviors have direct effects on blood sugar levels (23, 54). Some studies have found that farmers with underlying diseases and exposure to certain chemicals are significantly associated with adult-onset diabetes (56).

Ethnicity, sex, age, education level, financial status, underlying disease, smoking, knowledge, health behaviors, and the HbA1c level were significantly associated with the QoL. Multivariable analysis showed a high positive correlation between knowledge and health behaviors and QoL (beta = 0.770 and 0.886, respectively). These findings are consistent with the concept of health awareness leading to improvement in self-care behaviors (40). The American Diabetes Association reported that the QoL is an indicator of psychosocial outcomes in DM control (57). Previous research had also indicated that people with DM had a lower QoL than those not suffering from DM (58). Some studies have suggested that people with good QoL may have high motivation to control blood sugar levels, which would result in lower HbA1c levels (23).

Those studies have also pointed out that control of HbA1c levels in DM patients requires knowledge that would enable them to have better self-care and behavioral modifications (23). Cross-sectional studies have shown that self-care behaviors influence QoL directly (59, 60). Several cross-sectional and longitudinal studies have indicated self-care behaviors to be significantly negatively associated with the HbA1c level (61, 62). The QoL of ethnic minority agricultural workers is essential to their wellbeing in terms of undertaking high-quality work and generating income for them (4, 43).

The perception of the person under the ethnic culture are associated with awareness of the individual in term of four components: physical, mental, social relationships, and environment, which these elements are interrelated (63). Each ethnic group has different backgrounds and lifestyles, and they are unique in each area. Ethnic minorities show their identity through culture, traditions, lifestyles, and beliefs which may be associated with their QoL (9, 10). One longitudinal study demonstrated that self-care behaviors may affect QoL directly among ethnic minorities during the first 6-months of study, and that self-care behaviors are significant for subsequent QoL (23). Some studies have suggested that health and QoL are linked to lifestyle and social factors (20).

According to the findings, both HbA1c levels and quality of life are considered to be influenced by health behaviors. HbA1c levels may play a role in mediating the link between health behavior and quality of life. Normally, HbA1C is used as a sugar control monitor and a quality-of-life indicator in diabetic patients. In this study, it is also used as a marker of undiagnosed DM. Poor health behaviors such as eating habits and exercising in different ethnic minorities are considered to produce health problems such as high HbA1C levels, hypertension, and dyslipidemia which then affect quality of life. Individuals should be encouraged to recognize their commitment to health behaviors. They would be able to control HbA1c levels if they focused on appropriate health behavior modifications as well as avoiding health risk factors. The importance of an abnormal HbA1C level and the need to access health services for diagnosis and treatment should be emphasized in particular for the risk group. Furthermore, there should be information available in ethnic dialects that allows them to quickly access health information in a format that is appropriate for them. This may improve the quality of life for ethnic minority agricultural workers in rural areas.

There were some limitations to our research. First, due to the second wave of the COVID-19 epidemic and an announcement by the Thailand government to limit the number of people gathering in an area, some people refused to participate in our study. Second, causal inferences are impossible to draw from a cross-sectional study. Third, because of the difficulty of communicating in a local language and the large number of research assistants conducting face-to-face conversations, the information obtained may not be comprehensive and may affect the quality of data. This study did not take into account occupational factors such as working environments. Further research should also examine if there is a correlation between agricultural workers use of pesticides and HbA1c level and health. Additional types of blood lipids should be studied to develop a model for modifying healthcare behaviors among ethnic minorities. Blood pressure and other variables together with physical examination should also be considered.

## Conclusion

BMI, having an underlying disease, smoking, and health behaviors were related to the HbA1c level, accounting for 24.6% of the variance. Education, health behaviors, and HbA1c level were associated with QoL. These three factors could explain 79.4% of the variance in QoL among ethnic minority agricultural workers. An intervention programs should be tailored to the local context, focusing on eating behaviors such as cooking and eating local foods, eating beliefs, unhealthy lifestyles, and avoiding risk factors that contribute to health problems. In this case, improving health behaviors is helpful for increasing HbA1C control and overall quality of life.

## Data Availability Statement

The original contributions presented in the study are included in the article/supplementary materials, further inquiries can be directed to the corresponding author/s.

## Ethics Statement

The studies involving human participants were reviewed and approved by the University of Phayao Human Ethics Committee, Thailand (UP-HEC-1.2/023/64). The patients/participants provided their written informed consent to participate in this study.

## Author Contributions

SK and SBoon contributed to the conception and design of the work. SBoot, NA, MC, DD, and PP contributed the data acquisition. PO-A and KS contributed the analysis and interpretation of data for the work. SK, SBoon, and PO-A contributed drafting the work. SK and PO-A contributed revising the work for important intellectual content. All authors approved of the final version to be published and agree to be accountable for all aspects of the work in ensuring that questions related to the accuracy or integrity of any part of the work are appropriately investigated and resolved.

## Funding

The authors are grateful to the research project was supported by School of Medicine (MD-65-04) and the Thailand Science Research and Innovation fund and the University of Phayao the Unit of Excellence.

## Conflict of Interest

The authors declare that the research was conducted in the absence of any commercial or financial relationships that could be construed as a potential conflict of interest.

## Publisher's Note

All claims expressed in this article are solely those of the authors and do not necessarily represent those of their affiliated organizations, or those of the publisher, the editors and the reviewers. Any product that may be evaluated in this article, or claim that may be made by its manufacturer, is not guaranteed or endorsed by the publisher.
